# Supplementary material for: Evolutionary Dynamics of Whole-Genome Influenza A/H3N2 Viruses Isolated in Myanmar from 2015 to 2019
Source: Viruses. 2022 Oct 31;14(11):2414. doi: 10.3390/v14112414 (PMC9699102; doi:10.3390/v14112414)
Supplement: Supplementary file 1 [file viruses-14-02414-s001.zip › Myanamr Flu NGS Supplementary figure.pdf]

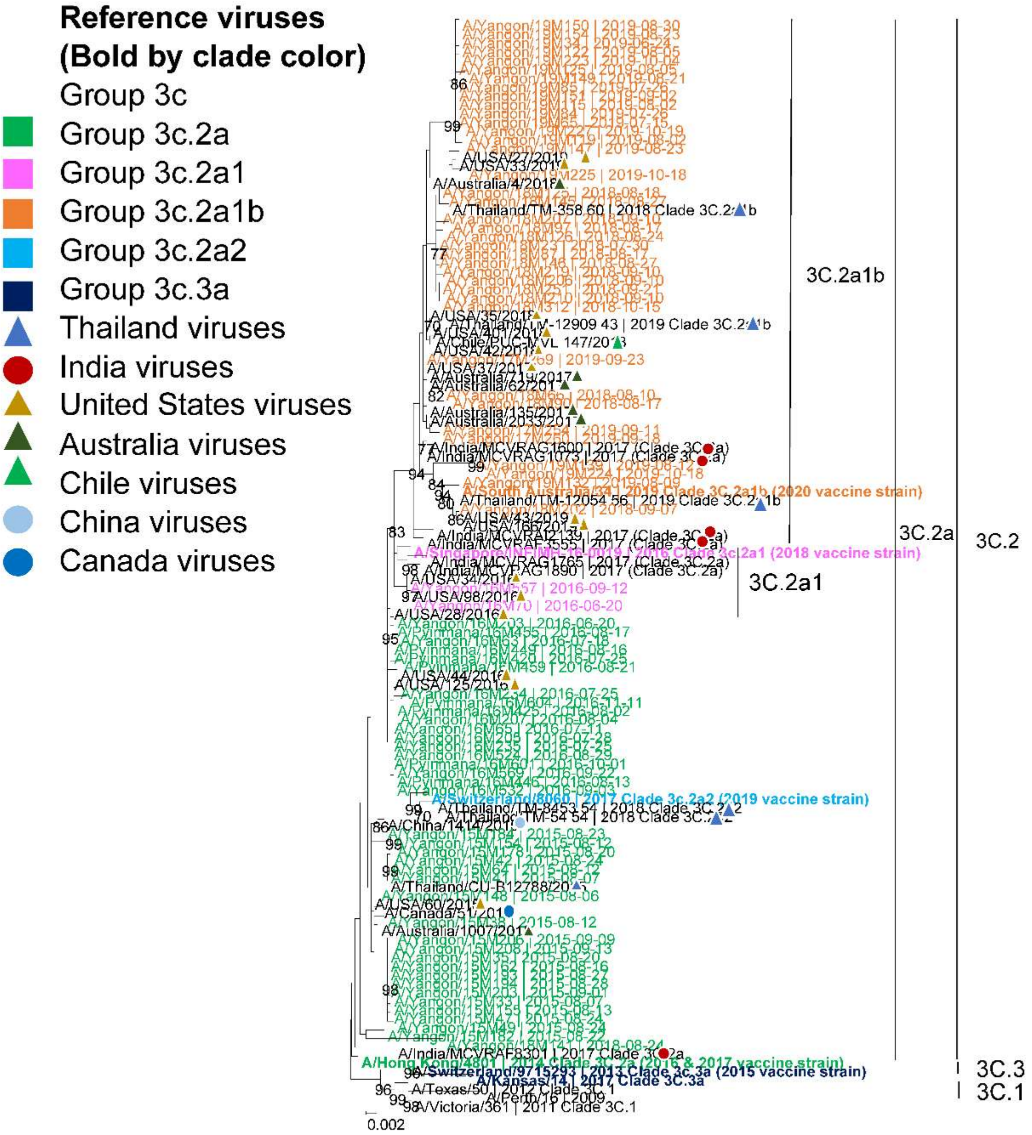

Supplementary Figure S1. Phylogenetic relationships of the HA gene segments of 125 sequences that comprise 79 A/H3N2 isolates from Myanmar (2015–2019) and the closest BLAST hits from different regions worldwide. The maximum-likelihood tree was inferred from 125 HA gene sequences in comparison with the Southern Hemisphere vaccine strains of known clades recommended by the World Health Organization (bold). Those in different colors represent Myanmar strains, colored according to clade. Those in black represent global strains. Branch values >70% are indicated at the nodes.
