# Supplementary material for: Evolutionary Dynamics of Whole-Genome Influenza A/H3N2 Viruses Isolated in Myanmar from 2015 to 2019
Source: Viruses. 2022 Oct 31;14(11):2414. doi: 10.3390/v14112414 (PMC9699102; doi:10.3390/v14112414)
Supplement: Supplementary file 1 [file viruses-14-02414-s001.zip › Supplementary Table S1 to S6.pdf]

Supplementary Table S1. List of mutation differences in the HA gene between Myanmar 2015–2019 viruses and the A/Texas/50/2012 vaccine strain.

Year	Clade	Amino acid mutations found in each position
2015	3C.2a	L3I, N144S, N145S, F159Y, K160T, N225D, Q311H, S144I, K450R
2016	3C.2a	L3I, N144S, N145S, F159Y, K160T, N225D, Q311H, S144I, K450R
	3C.2a1	L3I, N128T, N144S, N145S, F159Y, K160T, N225D, Q311H, N171K
2017	3C.2a1b	L3I, N128T, N144S, N145S, F159Y, K160T, N225D, E62G, K92R, N121K, T135K, R142G, N171K
2018	3C.2a	L3I, N144S, N145S, F159Y, K160T, N225D, Q311H, S144I, K450R, Q311H
	3C.2a1b	L3I, N144S, N145S, F159Y, K160T, N225D, E62G, K92R, N121K, T135K, R142G, N171K
2019	3C.2a1b	L3I, N144S, N145S, F159Y, K160T, N225D, E62G, K92R, N121K, T135K, R142G, N171K

Supplementary Table S2. List of mutation differences in the NA gene between Myanmar 2015–2019 viruses and the vaccine strains in respective seasons.

Representative strain	Clade	Amino acid mutations in each nucleotide position*												
2015		245	247	267	380	392								
A/Switzerland/9715293/2013	3C.3a	S	S	T	I	T								
2015 Myanmar viruses	3C.2a	N	T	K	V	I								
2016		140	231	245	247	267	339	380	392	468				
A/Hong Kong/4801/2014	3C.2a	L	I	S	S	T	D	I	T	P				
2016 Myanmar viruses	3C.2a and 3C.2a1	I	V	N	T	K	N	V	I	H				
2017		75	126	220	231	245	247	267	303	329	339	380	392	468
A/Hong Kong/4801/2014	3C.2a	K	P	K	I	S	S	T	V	N	D	I	T	P
2017 Myanmar viruses	3C.2a1b	R	L	N	V	N	T	K	I	S	N	V	I	H
2018		126	212	220	303	329								
A/Singapore/INFIMH-16-0019/2016	3C.2a1	P	I	K	V	N								
2018 Myanmar viruses	3C.2a and 3C.2a1b	L	V	N	I	S								
2019		126	176	194	220	303	344	386	469					
A/Switzerland/8060/2017	3C.2a2	P	M	V	K	V	E	S	I					
2019 Myanmar viruses	3C.2a1b	L	I	I	N	I	K	P	T					

*Amino acid mutations are compared to the vaccine strains in each season from 2015 to 2019.

Supplementary Table S3. List of mutation differences in the PB2 gene between Myanmar 2015–2019 viruses and the vaccine strains in respective seasons.

Representative strain	Clade	Amino acid mutations in each nucleotide position*						
2015		63	299	354	588			
A/Switzerland/9715293/2013	3C.3a	V	R	I	I			
2015 Myanmar viruses	3C.2a	I	K	V	T			
2016		63	299	470	588			
A/Hong Kong/4801/2014	3C.2a	V	R	S	I			
2016 Myanmar viruses	3C.2a and 3C.2a1	I	K	N	T			
2017		63	107	340	588	684		
A/Hong Kong/4801/2014	3C.2a	V	S	K	I	S		
2017 Myanmar viruses	3C.2a1b	I	N	R	T	P		
2018		96	107	299	451	340	588	545
A/Singapore/INFIMH-16-0019/2016	3C.2a1	I	S	K	V	K	I	V
2018 Myanmar viruses	3C.2a and 3C.2a1b	V	N	R	I	R	T	I
2019		107	147	299	340	410	444	
A/Switzerland/8060/2017	3C.2a2	S	T	K	K	M	V	
2019 Myanmar viruses	3C.2a1b	N	I	R	R	V	I	

*Amino acid mutations are compared to the vaccine strains in each season from 2015 to 2019.

Supplementary Table S4. List of mutation differences in the PB1 gene between Myanmar 2015–2019 viruses and the vaccine strains in respective seasons.

Representative strain	Clade	Amino acid mutations in each nucleotide position*			
2015		155	753	507	
A/Switzerland/9715293/2013	3C.3a	L	L	M	
2015 Myanmar viruses	3C.2a	F	I	T	
2016		152	166	216	668
A/Hong Kong/4801/2014	3C.2a	T	Y	G	P
2016 Myanmar viruses	3C.2a and 3C.2a1	S	F	C	L
2017		152	166		
A/Hong Kong/4801/2014	3C.2a	T	Y		
2017 Myanmar viruses	3C.2a1b	S	F		
2018		56	200	574	667
A/Singapore/INFIMH-16-0019/2016	3C.2a1	T	V	S	I
2018 Myanmar viruses	3C.2a and 3C.2a1b	A	I	L	N
2019		52	383		
A/Switzerland/8060/2017	3C.2a2	R	E		
2019 Myanmar viruses	3C.2a1b	K	D		

*Amino acid mutations are compared to the vaccine strains in each season from 2015 to 2019.

Supplementary Table S5. List of mutation differences in the PA gene between Myanmar 2015–2019 viruses and the vaccine strains in respective seasons.

Representative strain	Clade	Amino acid mutations in each nucleotide position*										
2015		256	272	308	407	534	554	605	668	669	675	713
A/Switzerland/9715293/2013	3C.3a	K	N	V	M	P	V	R	V	I	N	Y
2015 Myanmar viruses	3C.2a	Q	S	I	I	T	I	K	I	V	K	H
2016		272	284	357	611	639						
A/Hong Kong/4801/2014	3C.2a	N	L	T	F	T						
2016 Myanmar viruses	3C.2a and 3C.2a1	S	M	A	L	I						
2017		158	251	727								
A/Hong Kong/4801/2014	3C.2a	K	R	V								
2017 Myanmar viruses	3C.2a1b	R	K	I								
2018		105	158	551	668							
A/Singapore/INFIMH-16-0019/2016	3C.2a1	L	K	R	V							
2018 Myanmar viruses	3C.2a and 3C.2a1b	F	R	K	I							
2019		158	626	648								
A/Switzerland/8060/2017	3C.2a2	K	K	S								
2019 Myanmar viruses	3C.2a1b	R	R	N								

*Amino acid mutations are compared to the vaccine strains in each season from 2015 to 2019.

Supplementary Table S6. List of mutation differences in the NP gene between Myanmar 2015–2019 viruses and the vaccine strains in respective seasons.

Representative strain	Clade	Amino acid mutations in each nucleotide position*			
		217			
A/Switzerland/9715293/2013	3C.3a	G			
2015 Myanmar viruses	3C.2a	S			
		101	417	454	472
A/Hong Kong/4801/2014	3C.2a	G	N	D	T
2016 Myanmar viruses	3C.2a and 3C.2a1	D	S	E	A
		101	197	454	472
A/Hong Kong/4801/2014	3C.2a	G	V	D	T
2017 Myanmar viruses	3C.2a1b	D	I	E	A
		197	421	450	472
A/Singapore/INFIMH-16-0019/2016	3C.2a1	V	E	G	A
2018 Myanmar viruses	3C.2a and 3C.2a1b	I	D	S	T
		220	363	418	
A/Switzerland/8060/2017	3C.2a2	E	V	L	
2019 Myanmar viruses	3C.2a1b	D	I	I	

*Amino acid mutations are compared to the vaccine strains in each season from 2015 to 2019.
